# Altered Perivascular Diffusivity in Glioblastoma:Integrating DTI-ALPS Index with Radio-Pathomicand Histopathologic Correlates

**DOI:** 10.21203/rs.3.rs-8290863/v1

**Published:** 2025-12-22

**Authors:** Biprojit Nath, Samuel A. Bobholz, Daniel C. Kim, Allison K. Lowman, Savannah R. Duenweg, Aleksandra Winiarz, Benjamin Chao, Fitzgerald Kyereme, Michael Barrett, Hope M. Reecher, Jennifer Connelly, E.Kelly S.Mrachek, J. Jacobsohn, Max O. Krucoff, Elaine T, A Mohit, D Daniel, Anjishnu Banerjee, Peter S. LaViolette

**Affiliations:** Medical College of Wisconsin; Medical College of Wisconsin; Medical College of Wisconsin; Medical College of Wisconsin; Medical College of Wisconsin; Medical College of Wisconsin; Medical College of Wisconsin; Medical College of Wisconsin; Medical College of Wisconsin; Medical College of Wisconsin; Medical College of Wisconsin; Medical College of Wisconsin; Medical College of Wisconsin; Medical College of Wisconsin; Medical College of Wisconsin; Medical College of Wisconsin; Medical College of Wisconsin; Medical College of Wisconsin; Medical College of Wisconsin

## Abstract

**Purpose:**

Glioblastoma is an aggressive primary brain tumor that often exhibits perivascular invasion. This behavior may directly interfere with glymphatic flow, hindering perivascular drainage routes. This study aims to assess glymphatic dysfunction in glioblastoma by evaluating the DTI-ALPS index, an MRI-based surrogate of glymphatic activity. We additionally correlate mpMRI-derived tumor features with radio-pathomic maps of hypercellularity.

**Methods:**

We included 368 IDH-wildtype GBM patients from the UCSF-PDGM dataset. Preoperative T1, T1C, FLAIR, ADC, and diffusion tensor imaging (DTI) maps were preprocessed using standard co-registration and intensity normalization protocols. Radio-pathomic maps of tumor cellularity were generated using a previously published model which was trained on spatially aligned autopsy samples. The DTI-ALPS index was computed using DTI maps normalized to the JHU-ICMB-FA template, with ROIs on predefined white matter tracts and categorized by tumor laterality.

**Results:**

The DTI-ALPS index was significantly lower on the ipsilateral side for both the GTR and STR cohorts (p < 0.00001). Furthermore, DTI-ALPS _mean_ and DTI-ALPS _ipsilateral_ showed an inverse association with contrast enhancing and FLAIR hyperintensity volumes (both p < 0.00001) and total cellularity within the contrast enhancing and FLAIR hyperintensity regions (both p < 0.00001). Notably, autopsy tissue analysis revealed SOX2 positive tumor cells in the perivascular spaces. Survival analysis further demonstrated that patients with lower DTI-ALPS _mean_ indices exhibited significantly worse overall survival (log-rank p < 0.05).

**Conclusion:**

Our findings suggest that GBM-associated perivascular invasion disrupts glymphatic function, as evidenced by a significantly lower DTI-ALPS index in the tumor-affected hemisphere. The inverse correlation between the DTI-ALPS index and both volume and cellularity metrics highlights the extent to which GBM alters perivascular fluid dynamics.

## 1. Introduction

Glioblastoma (GBM) is the most common primary brain tumor in adults [1]. It is characterized by rapid proliferation [1, 2] and resistance to therapy [4–10], and it has a median overall survival of 14.6–20.9 months even with standard of care therapy [11, 12]. While much research has focused on the tumor’s aggressive nature and treatment challenges, growing evidence suggests that GBM affects key physiological processes beyond the tumors’ infiltrative boundaries, such as the brain’s glymphatic waste clearance mechanism [13, 14]. The glymphatic system is a brain-wide pathway that facilitates waste clearance by promoting convective exchange between cerebrospinal fluid (CSF) and interstitial fluid (ISF) aided by aquaporin-4 (AQP4) channels on astrocyte end-feet surrounding the perivascular spaces [15]. Its dysfunction may contribute to the accumulation of toxic metabolites and further exacerbate the tumor microenvironment, which has been implicated in other neurological diseases such as Alzheimer’s [16], Parkinson’s disease[17], and traumatic brain injury[18].

To assess glymphatic alterations noninvasively, imaging-based metrics have recently been explored [19]. One such metric is the diffusion tensor image (DTI) along the perivascular space index (DTI-ALPS), which quantifies water diffusivity [19]. This method has been used as a putative marker of glymphatic function in neurodegenerative diseases, but its relevance in GBM remains less explored [20, 21].

In GBM, microscopic tumor infiltration often extends beyond contrast-enhancing regions [22, 23]. Coupled with vascular abnormalities, tumor presence may alter perivascular fluid dynamics. To map tumor invasion beyond contrast enhancement, we have previously developed a radio-pathomic algorithm using autopsy-validated GBM specimens [24–27]. This multistage framework aligns histological annotations with pre-mortem multiparametric MRI (mpMRI) to train a machine learning model that estimates cellularity, extracellular fluid, and cytoplasm density, as well as voxel-wise tumor probability based on imaging features. These models have demonstrated the ability to detect non enhancing, infiltrative tumor associated with poorer survival in independent cohorts [25, 26].

This study integrates DTI-ALPS metrics, mpMRI based features, and radio-pathomic maps of cell density to characterize glymphatic dysfunction in GBM. We hypothesize that glioblastoma induced alterations, such as increased cellularity and tumor infiltration beyond contrast enhancing regions may disrupt perivascular fluid transport reflected by lower DTI-ALPS indices that serve as an indirect marker of glymphatic function.

We additionally show examples of glymphatic invasion as seen at autopsy compared to radio-pathomic maps for comparison.

## 2. Methods

### 2.1 Patient population

DTI-ALPS: The publicly available imaging UCSF-Preoperative Diffuse Glioma MRI dataset [28] which consisted of 501 adult patients with pathologically confirmed WHO grade 2–4 diffuse gliomas, based on the 2021 WHO classification [29]. Inclusion criteria for this study required IDH-wildtype GBM diagnosis, resulting in 368 adult patients included.

Autopsy: One representative case from the Medical College of Wisconsin Neuro-oncology brain bank was assessed to determine prevalence and extent of perivascular invasion compared to imaging findings from the final mpMRI exam prior to death.

### 2.2 MR Imaging Acquisition and Preprocessing

Imaging data for this study was acquired following standard clinical imaging protocols established at UCSF with detailed information regarding the imaging protocols available [28]. Our study specifically assessed T1, T1 post-contrast (T1C), T2-weighted fluid attenuated inversion recovery (FLAIR), apparent diffusion coefficient (ADC) maps calculated from diffusion weighted imaging (DWI), diffusion tensor imaging (DTI) derived maps of fractional anisotropy (FA), and diffusivity maps along x, y and z directions (d_xx,_ d_yy_, d_zz_ maps). Multicompartment tumor segmentation (necrotic core, contrast enhancing (CE) tumor, and peritumoral edema) using semi-automated, radiologist-corrected masks were additionally provided in the dataset [28, 30].

### 2.3 Radio-pathomic mapping

The MRIs from each patient were used to generate radio-pathomic maps of cellularity using a pre-established algorithm [24, 26, 31]. Briefly, the T1, T1C, and ADC images were rigidly registered to the FLAIR images using SPM12 (https://www.fil.ion.ucl.ac.uk/spm/software/spm12/). Images were intensity normalized (T1, T1C and FLAIR) by dividing by the standard deviation of intensity within a mask of the brain on each respective image. This multimodal input was then fed to a bagged random forest machine learning algorithm trained on a separate cohort with spatially-aligned autopsy samples. This resulted in voxel-wise predictions of tumor cellularity, extracellular fluid, and cytoplasm density based on mpMRI features [24, 26, 27]. These maps have previously been found to detect areas of pathologically validated hypercellular tumor beyond the contrast-enhancing margin and beyond the FLAIR hyperintensity in some cases. [18, 25, 26]

### 2.4 DTI-ALPS calculation

The Diffusion Tensor Imaging–Analysis Along the Perivascular Space (DTI-ALPS) index was computed using eddy current–corrected diffusion tensor maps (d_xx_, d_yy_, and d_zz_) provided from the dataset [28]. To standardize region of interest (ROI) placement and avoid subject-specific variability, all DTI maps were spatially normalized to the JHU-ICMB-FA template space (https://identifiers.org/neurovault.image:1402) using advanced normalization tools (ANTs) [32]. The ROIs required for ALPS computation were adopted from the method described by Liu et al. [33], where ROIs are predefined in major white matter tracts such as the superior longitudinal fasciculus (SLF) and the superior corona radiata (SCR). This template-based approach eliminated the need for manual ROI delineation, ensuring consistent and reproducible measurements across subjects.

The DTI-ALPS index was calculated separately for both the left and right hemispheres, and the resulting values were labeled as DTI-ALPS _left_ and DTI-ALPS _right_, respectively. To incorporate the spatial relationship between the tumor and the DTI-ALPS computation, each index was further categorized as DTI-ALPS _ipsilateral_ or DTI-ALPS _contralateral_, depending on whether the measurement was taken from the hemisphere containing the tumor or the opposite hemisphere. Additionally, the DTI-ALPS _mean_ was computed as the average of the left and right indices to provide a summary measure of overall glymphatic function.

### 2.5 Histopathological validation

Autopsy brain tissue from a representative patient from the MCW Neuro-Oncology brain bank was included for histopathological analysis in relation to imaging-derived glymphatic measures. The patient was a 59-year-old male with IDH-wildtype GBM, treated with temozolomide, bevacizumab, pulsed low-dose radiation (PLDR), and lomustine (CCNU), with an overall survival of 1,601 days, representing a long-term survivor that enabled assessment of post-treatment and microenvironmental changes associated with glymphatic dysfunction. The final MRI was acquired 16 days prior to death and included diffusion tensor imaging (DTI) sequences relevant for DTI-ALPS index calculation. Perivascular and adjacent tumor regions were reviewed by a board-certified neuropathologist. SOX2 immunohistochemistry (IHC) was used to identify tumor cells, as it is frequently over-expressed in glioblastoma, including in subpopulations with stem-like properties [34, 35].

### 2.6 Statistical analysis

Paired t-tests were used to compare DTI-ALPS metrics between group comparisons. For assessing associations between DTI-ALPS metrics and imaging-derived tumor features (CE volume, FLAIR hyperintensity volume, and CE total cellularity), with separate linear regression analyses were conducted for each group based on extent of resection (i.e., GTR, STR). Regression outputs included estimated coefficients, R^2^, and p-value, with results considered significant at p < 0.05. For survival analysis, patients were dichotomized into high and low DTI-ALPS groups using a median split. Kaplan–Meier survival curves were generated, and differences between groups were evaluated using the log-rank test.

## 3. Results

### 3.1 DTI-ALPS index and tumor laterality

**Figure 2:**
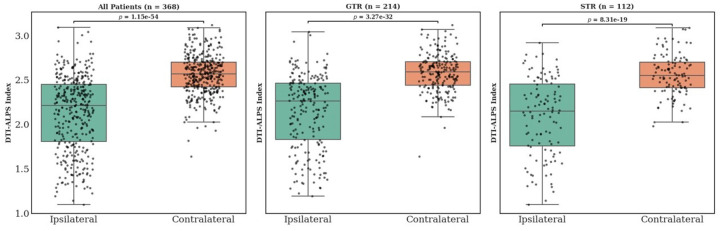
Comparison of DTI-ALPS index between ipsilateral and contralateral hemispheres. Boxplots show significantly lower DTI-ALPS _ipsilateral_ values compared to DTI-ALPS _contralateral_ across all patients (left panel), patients who underwent gross total resection (GTR, middle panel), and those who underwent subtotal resection (STR, right panel). Each dot represents an individual patient, and p-values are based on paired statistical comparisons.

The DTI-ALPS index was significantly reduced in the hemisphere ipsilateral to the tumor compared to the contralateral hemisphere. For the entire cohort (n = 368), the ipsilateral DTI-ALPS index was 2.131 ± 0.4173 versus 2.561 ± 0.225 on the contralateral side (t=−18.55, p < 0.00001). For patients who underwent GTR, the ipsilateral versus contralateral ALPS index was 2.150 ± 0.414 vs. 2.578 ± 0.215 (t=−14.064, p < 0.00001). Similarly, in patients with STR, the ALPS index was 2.088 ± 0.434 vs. 2.558 ± 0.231 (t=−10.708, p < 0.00001). Box plots showing the differences are shown in Fig. 2.

### 3.2 Correlation between DTI-ALPS index, CE tumor volume, and FLAIR hyperintensity volume

**Figure 3:**
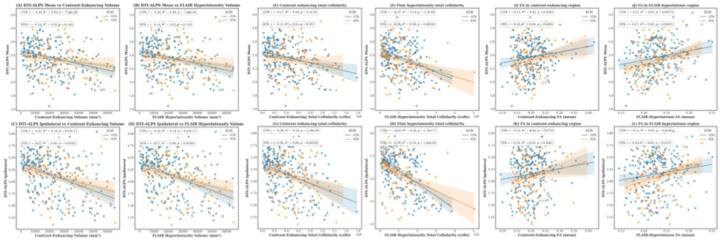
Associations between DTI-ALPS indices and imaging features derived from mpMRI. Scatter plots show linear regression relationships between DTI-ALPS _mean_ (top row) and DTI-ALPS _ipsilateral_ (bottom row) with various imaging features derived from mpMRI across all patients and stratified by extent of resection (GTR vs STR). (A–D) Higher contrast-enhancing and FLAIR hyperintensity volumes are associated with reduced DTI-ALPS indices. (E–H) Increased total cellularity within contrast-enhancing and FLAIR hyperintensity regions shows significant inverse correlations with both ALPS indices. (I–L) Mean FA within contrast-enhancing and FLAIR-defined hyperintense regions shows a positive correlation with DTI-ALPS indices.

An inverse correlation was observed between the DTI-ALPS _ipsilateral_ index and CE tumor volume (r = − 0.42, p < 0.00001 for GTR; r = − 0.27, p = 0.003 for STR). A similar association was noted for the DTI-ALPS _mean_ index (r = − 0.30, p < 0.0001 for GTR; r = − 0.14, p = 0.14 for STR). We also found a significant inverse correlation between the DTI-ALPS _ipsilateral_ index and FLAIR-defined hyperintensity volume (r = − 0.42, p < 0.00001 for GTR; r = − 0.30, p < 0.00001 for STR), with a similar association for the DTI-ALPS _mean_ index (r = − 0.30, p < 0.0001 for GTR; r = − 0.14, p = 0.143 for STR). Scatter plots illustrating the correlations are shown in Fig. 3 (A–D).

### 3.3 Correlation between DTI-ALPS index and fractional anisotropy

A positive correlation was observed between the DTI-ALPS _ipsilateral_ index and Fractional anisotropy within the CE tumor region in both GTR/STR groups, though only significant in STR (r = 0.12, p = 0.07 for GTR; r = 0.18, p = 0.05 for STR). A similar association was found for the DTI-ALPS _mean_ (r = 0.15, p = 0.02 for GTR; r = 0.20, p = 0.03 for STR). Additionally, we observed a similar positive correlation between DTI-ALPS _ipsilateral_ index and Fractional anisotropy within the FLAIR defined hyperintense region (r = 0.12, p = 0.07 for GTR; r = 0.18, p = 0.05 for STR). Similarly, positive correlations were seen for DTI-ALPS _mean_ (r = 0.21, p = 0.001 for GTR; r = 0.27, p = 0.004). Scatter plots illustrating the correlations are shown in Fig. 3 (I–L).

### 3.4 Correlation between DTI-ALPS Index and predicted cellularity in regions of CE and peritumoral edema

An inverse correlation was observed between the DTI-ALPS _ipsilateral_ index and total cellularity within the CE tumor region (r = − 0.39, p < 0.00001 for GTR; r = − 0.28, p = 0.003 for STR). A similar association was found for the DTI-ALPS _mean_ index (r = − 0.28, p < 0.0001 for GTR; r = − 0.18, p < 0.05 for STR). These associations were also observed within the FLAIR-defined hyperintensity region, where both DTI-ALPS _ipsilateral_ and DTI-ALPS _mean_ indices showed significant negative correlations with total cellularity. Scatter plots illustrating the correlations are shown in Fig. 3 (E–H).

### 3.5 DTI-ALPS Index and Corresponding Findings from Brain Autopsy Samples

In the representative patient with postmortem tissue available, the DTI-ALPS index in the tumor-affected hemisphere was markedly reduced (DTI-ALPS_ipsilateral_ = 1.16) compared to the contralateral hemisphere (DTI-ALPS_contralateral_ = 1.37). Corresponding autopsy analysis revealed prominent perivascular infiltration by SOX2-positive tumor cells (Fig. 5), suggesting that perivascular tumor cells infiltration may have contributed to reduced DTI-ALPS index.

### 3.6 Prognostic value of DTI-ALPS index on overall survival

**Figure 4:**
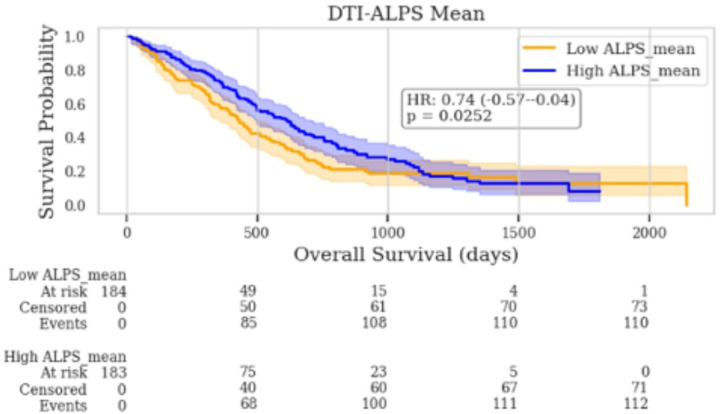
Overall survival stratified by DTI-ALPS indices. Kaplan–Meier survival curves show overall survival probability for patients grouped by median split into high vs. low DTI-ALPS values for (left) DTI-ALPS _mean_.

Patients with lower DTI-ALPS _mean_ had significantly shorter overall survival compared to those with higher DTI-ALPS _mean_ (446 versus 591 days, log-rank p-value = 0.02, HR = 0.74). DTI-ALPS _ipsilateral_ (log-rank p-value = 0.19, HR = 0.84) and DTI-ALPS _contralateral_ (log-rank p-value = 0.50, HR = 0.91) were not significantly associated with survival when analyzed individually (Fig. 4).

## 4. Discussion

This study investigates glymphatic function in GBM using the DTI-ALPS index, a non-invasive imaging biomarker that reflects water diffusivity along the perivascular spaces. We observed a significantly reduced DTI-ALPS index in the tumor-bearing (ipsilateral) hemisphere compared to the contralateral side. This hemispheric disparity was observed consistently across all patients who underwent both GTR and STR. Also, we observed greater impairment in DTI-ALPS index in patients with larger CE tumor volumes, extensive FLAIR hyperintensity, and increased cellularity in the contrast enhancing and FLAIR hyperintensity regions. Conversely, higher fractional anisotropy (FA) in tumor and peritumoral regions were associated with higher DTI-ALPS index. While DTI-ALPS does not directly measure glymphatic function and can be influenced by edema or tissue anisotropy, it isolates directional diffusivity along perivascular pathways by comparing it to orthogonal directions near the ventricles [19, 36]. This normalization reduces sensitivity to isotropic edema effects, making ALPS a useful proxy for perivascular transport. The observed reductions suggest that cellular and edematous disruption may hinder glymphatic-like flow.

Several pathophysiological mechanisms could underlie the pretreatment glymphatic dysfunction observed in GBM patients. First, physical obstruction of perivascular pathways by infiltrating tumor cells is a likely contributor. Invasive glioma cells preferentially migrate along white matter tracts and perivascular spaces, often encircling the blood vessels [37]. Perivascular invasion disrupts the blood-brain barrier by separating the astrocyte end feet from blood vessels [38]. Second, tumor-associated edema and elevated interstitial pressure in gliomas may further impair glymphatic function [39]. Third, the mass effect from expanding solid tumor generates solid stress and can mechanically compress the microscopic pathways of the glymphatic system [40]. Finally, AQP4 dysregulation is also a contributing factor for impaired glymphatic clearance [41, 42]. Together, these mechanisms likely contribute to the decreased DTI-ALPS indices observed in the glioma patients.

**Figure 5:**
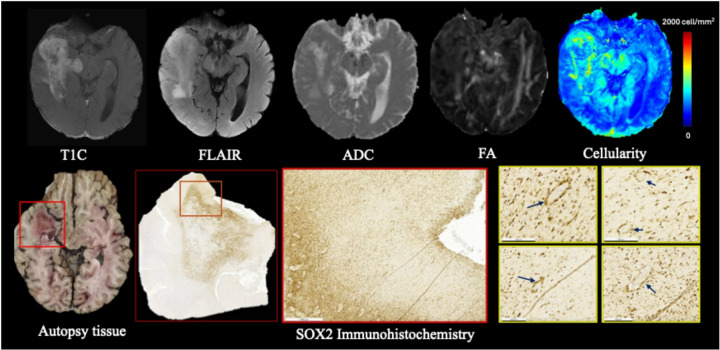
Multimodal imaging and histopathologic validation of tumor cellularity near perivascular regions. Top row: Axial MRI sequences (T1C, FLAIR, ADC, FA) and radiopathomic cellularity map from a representative GBM patient, highlighting elevated cellularity in tumor-infiltrated regions. Bottom row: Corresponding autopsy brain tissue and SOX2 immunohistochemistry from the same region (outlined in red) confirm tumor presence. High-magnification SOX2-stained images (yellow boxes) reveal SOX2 tumor cells concentrated around perivascular spaces (blue arrows), supporting imaging-based observations of glymphatic pathway disruption.

This study builds upon prior research into glymphatic function in gliomas using the DTI-ALPS index. Toh et al. (2021) were among the first to apply the ALPS index in a large glioma patient cohort (n = 201) [43]. They reported that IDH-wildtype gliomas had significantly lower DTI-ALPS indices than IDH-mutant gliomas, and larger volumes of peritumoral edema were independently associated with a lower DTI-ALPS index. We likewise found high-grade features of glioma, such as increased cellularity in the contrast enhancing and FLAIR hyperintense region, large contrast enhancing mass, large peritumoral edema volume, corresponds to poor DTI-ALPS index. Toh et al. speculated that glymphatic impairment could be a factor in edema pathogenesis [37]. Our work reinforces this hypothesis by demonstrating this relationship on a continuous scale (i.e., DTI-ALPS versus CE total cellularity). We additionally detected evidence of this process in postmortem tissue (Fig. 5), where SOX2-positive cells were observed infiltrating along blood vessels within the perivascular spaces near peritumoral locations. This pathological finding illustrates how glial cell invasion can physically impede the glymphatic conduits that normally facilitate cerebrospinal fluid–interstitial fluid (CSF–ISF) exchange.

More recently, Zeng et al. (2024)[44] conducted a study with diffuse glioma patients (n = 91) and healthy controls (n = 59), having found the DTI-ALPS_ipsilateral_ index to be lower than the contralateral, regardless of tumor grade, size, or molecular type, which is in agreement with our intra-patient hemispheric comparisons. Notably, Zeng et al. reported that patients with worse glymphatic function had shorter overall survival. Our study did see a similar association, with patients with lower DTI-ALPS _mean_ having significantly poorer survival outcomes compared to higher DTI-ALPS _mean_. Further, the observed correlation between DTI-ALPS and cellularity aligns with the idea that glymphatic dysfunction is present in higher-grade, malignant tumor phenotypes. We focused on the pretreatment period and intra-patient hemispheric comparisons, which eliminated inter-patient variability by using each patient’s contralateral hemisphere as an internal control. Both studies indicate that ipsilateral glymphatic dysfunction is a robust phenomenon in gliomas, and contralateral glymphatic disturbances may occur in advanced disease. Similar findings have also been reported by Villacis et al. on a smaller patient cohort (n = 24) [45].

Two recent studies further support glioma-related glymphatic dysfunction. Liang et al. (2025) [46] analyzed 112 patients with glioma and found significantly lower DTI-ALPS indices compared to healthy controls, along with increased total CSF volume. Their multivariate analysis identified tumor grade as an independent predictor of glymphatic impairment. Similarly, Tian et al. (2025) [47] reported globally reduced DTI-ALPS indices in high-grade glioma (HGG) patients, with the ipsilateral hemisphere most affected. These findings align closely with our results and those of Zeng et al., reinforcing the role of glymphatic dysfunction in glioma. Our study extends this evidence by demonstrating hemisphere-specific changes and providing histopathological validation of perivascular invasion. Animal models provide corroborating evidence; in a rat glioma model, the implanted tumor was shown to block the arterial perivascular influx pathway and downregulate AQP4 expression, resulting in diminished glymphatic tracer transport [48]. These findings align with our observation that hypercellular tumor regions (CE and peritumoral edema) exhibit reduced DTI-ALPS indices, likely reflecting a combination of structural and functional hindrance. Our study is among the first to incorporate imaging with ex vivo histology in this context, strengthening the interpretation that GBM compromises glymphatic pathways. The DTI-ALPS index may serve as a non-invasive imaging marker reflecting glioblastoma aggressiveness. While still investigational, glymphatic metrics could eventually aid in preoperative risk stratification and guide supportive management.

This study has several limitations that warrant consideration. First, the DTI-ALPS index is an indirect measure of glymphatic function [49]. However, this metric can be influenced by many microstructural factors like such as tumor-associated demyelination, necrosis, or increased free water from edema and thus reduce the DTI-ALPS index, even without changes in CSF flow. We attempted to mitigate this by computing an average DTI-ALPS index from both hemispheres; yet it is important to note that DTI-ALPS index changes are not direct measures, or reflective of CSF clearance. Second, our analysis did not include a healthy control group. While other prior studies have established that glioblastoma patients on average have lower DTI-ALPS indices than controls [39, 46], future studies should incorporate matched controls to delineate the degree of glymphatic dysfunction occurs in glioblastoma patients. Third, tumor cellularity in this study was estimated using a radio-pathomic model trained on autopsy-derived histology and co-registered MRI. While this model has been previously validated and offers voxel-wise biological insight, it remains a computational inference subject to spatial resolution constraints and model bias.Although we observed associations between reduced DTI-ALPS, tumor cellularity, and edema cellularity, we did not quantify contrast-enhancing (CE) versus peri-enhancing tumor burden, nor differentiate peri-CE perivascular from parenchymal invasion. Lastly, our analysis is cross-sectional, and the temporal evolution of DTI-ALPS indices in response to treatment or tumor progression could not be assessed. Future longitudinal studies integrating direct CSF imaging methods, dynamic physiological measurements, and post-treatment imaging are needed to confirm and extend these findings. Despite these limitations, our study provides strong initial evidence of glymphatic dysfunction in untreated glioma patients. It sets the stage for prospective studies to further elucidate this phenomenon and to explore whether improving glymphatic function can translate into clinical benefit for patients with gliomas.

## 5. Conclusion

Our study suggests that glioblastoma alters perivascular diffusivity within the tumor-affected hemisphere, reflected by significantly reduced DTI-ALPS indices. These reductions correlate with key tumor characteristics, being larger CE volumes, greater FLAIR hyperintensity, and increased cellularity in the CE and FLAIR hyperintensity regions, indicating that glymphatic impairment reflects underlying tumor aggressiveness. Importantly, we provide histopathological validation of this mechanism through autopsy findings showing perivascular infiltration by SOX2-positive tumor cells.

## Figures and Tables

**Figure 1:**
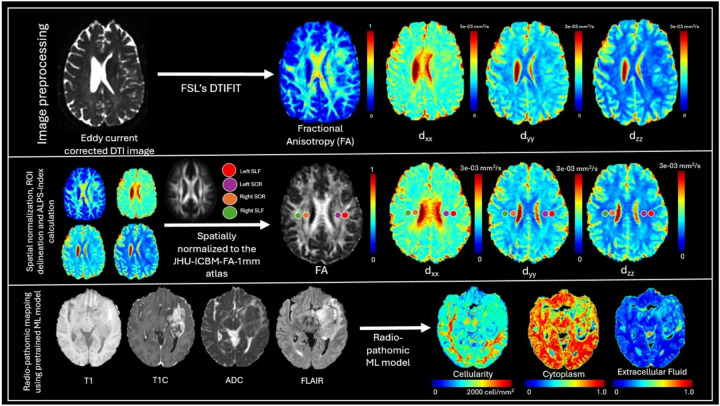
Pipeline for DTI-ALPS index computation and radiopathomic feature extraction. Top panel: Preprocessing of diffusion tensor imaging (DTI) data includes eddy current correction followed by tensor fitting using FSL’s DTIFIT to generate maps of fractional anisotropy (FA) and principal diffusivities (d_xx_, d_yy_, d_zz_). Middle panel: All diffusion maps are spatially normalized to the JHU-ICBM-FA-1mm atlas. Regions of interest (ROIs) in the left and right superior longitudinal fasciculus (SLF) and superior corona radiata (SCR) are delineated for DTI-ALPS index computation. Bottom panel: Structural MRI sequences (T1, T1C, ADC, FLAIR) are input into a pretrained machine learning model to derive voxel-wise maps of cellularity, cytoplasm, and extracellular fluid as part of radiopathomic modelling.

## Abbreviations

DTI-ALPS: Diffusion tensor imaging along the perivascular space
GTR: Gross total resection
STR: Subtotal resection
ROI: Region of interest
CE: Contrast enhancing
